# The Impact of Personal Gender-Typicality and Partner Gender-Traditionality on Taking Sexual Initiative: Investigating a Social Tuning Hypothesis

**DOI:** 10.3389/fpsyg.2017.00107

**Published:** 2017-02-01

**Authors:** Peggy M. J. Emmerink, Regina J. J. M. Van Den Eijnden, Tom F. M. Ter Bogt, Ine Vanwesenbeeck

**Affiliations:** ^1^Department of Interdisciplinary Social Sciences, Utrecht UniversityUtrecht, Netherlands; ^2^Rutgers Expert Centre for Sexual and Reproductive Health and RightsUtrecht, Netherlands

**Keywords:** assertiveness, experimental methods, gender identity, individual differences, social norms

## Abstract

Sexual assertiveness is an issue of interest in the context of gender equality and sexual health. This study investigated the social tuning hypothesis that encountering a gender-traditional partner would lead to stronger gender-typical behavior, i.e., respectively, higher and lower levels of taking sexual initiative among men and women. Participants (*N* = 271) read a vignette describing a romantic partner, who was either presented as gender-traditional or not, followed by a sexual scenario. Subsequently, participants were asked about their expectations toward their own sexual initiative taking. Results showed a significant ‘target gender-traditionality × participant gender × participant gender-typicality (masculinity/femininity)’ interaction meaning that less gender-typical men were more likely to initiate sexual contact in the experimental, compared to the control condition. Men low in masculine characteristics showed higher initiative taking in response to a gender-traditional target female. We conclude that less gender-typical men seem to employ more social tuning toward their sexual partner, whereas more gender-typical men seem to adhere to their gender-typical behavior regardless of perceived partner characteristics. These results were not seen among the women in the sample. These findings are a starting point for the further development of experimental investigations regarding the gendered nature of both sexual initiative taking and sexual assertiveness in general.

## Introduction

Sexual assertiveness remains an issue of great interest in the context of gender equality, sexual consent vs. aggression (Burkett and Hamilton, 2012), and overall young peoples’ sexual health (Crawford and Popp, 2003; Sanchez et al., 2012; Vanwesenbeeck, 2014). A lack of sexual assertiveness has for instance been related to poor sexual protection (Impett et al., 2006; Crosby et al., 2008), heightened body objectification (Impett et al., 2006; Ramsey and Hoyt, 2015), increased risk of sexual victimization (Zerubavel and Messman-Moore, 2013; Kelley et al., 2015), and poor sexual function (Kiefer et al., 2006; Sanchez et al., 2006).

Although different operationalisations of sexual assertiveness exist in the literature (Morokoff et al., 1997; Santos-Iglesias et al., 2014), and there is notable overlap with other concepts such as sexual autonomy (Sanchez et al., 2006) and sexual agency (Crosby et al., 2008; Burkett and Hamilton, 2012; Bay-Cheng, 2015), we understand sexual assertiveness in this study as ‘the ability to initiate desired sexual contacts, refuse unwanted sexual contacts, and the ability to prevent pregnancy or STIs’ (Santos-Iglesias et al., 2014, p. 232). In this study we chose to focus on sexual initiative taking, because research has time and again shown a pattern of relatively frequent sexual initiative by men and relatively frequent sexual compliance by women (Morokoff et al., 1997; Vannier and O’Sullivan, 2011; Sanchez et al., 2012), even in relatively sexually liberal cultures such as in the Netherlands (Schalet, 2010; De Graaf et al., 2012). These gendered differences in taking sexual initiative can in part be considered a result of the sexual double standard (SDS); ‘A traditional gender norm promoting sexual modesty for girls and women, but sexual prowess for boys and men’ (Emmerink et al., 2016a, p. 1). Already in first-time sexual encounters we see evidence of female submission and male dominance, attesting to the fact that gender norms influence sexual behavior from the very start of sexual exploration (Sanchez et al., 2012). Although adhering to gender norms can yield psychosocial benefits, such as increased self-esteem, feelings of social competence and peer acceptance (Smith and Leaper, 2006; Kreager and Staff, 2009; DiDonato and Berenbaum, 2012; Jewell and Brown, 2014), it also poses challenges to identity development and (sexual) health and wellbeing for young men and women (Crawford and Popp, 2003; Sanchez et al., 2012; Vanwesenbeeck, 2014). Review studies indicate that traditional gender roles are harmful for sexual expression both among men (Sanchez et al., 2012) and even more so among women (Crawford and Popp, 2003; Sanchez et al., 2012; Emmerink et al., 2016b). Moreover, these studies conclude that this can be at least partly explained by the sexually sub-assertive role that is most commonly seen among (young) women.

However, whereas reviews support the enduring influence of the SDS, they also stress that its endorsement and enactment are heavily influenced by cultural, situational, and interpersonal factors (Crawford and Popp, 2003; Fugère et al., 2008; Sanchez et al., 2012; Bordini and Sperb, 2013). Others have also noted that ‘the SDS seems to be a contextual phenomenon’ (Zaikman and Marks, 2014, p. 2; Zaikman and Marks, 2016). A framework that is of particular interest in this regard is Deaux and Major’s (1987) Interactive Model of Gender-Related Behavior. This model captures both the flexibility and context-dependency of gender norms that have been stressed in review studies on the SDS. Furthermore, Deaux and Major’s notion of gender differences as a case of ‘now you see them, now you don’t’ seems particularly applicable in the field of SDS research, which has been troubled by mixed findings in the past (Sanchez et al., 2012). This leads to the conclusion that in order to further the knowledge on how SDSs continue to shape (adolescent) sexuality, innovative research in this field needs to take into account the role of cultural embeddedness, situational factors, and interpersonal processes.

The present study tries to accommodate this notion by investigating whether gender differences in sexual initiative taking (in line with the SDS) can be explained through social tuning, as proposed by Deaux and Major (1987). Social tuning refers to the tendency of individuals to conform to perceived (explicit and implicit) norm and expectation signals sent out by interaction partners. Individuals then “tune” their social beliefs and subsequent behavior toward the views of the other (Sinclair et al., 2005). Gendered attitudes and behavior are also involved in these processes. Because we expect to find SDSs in the views and behavior of others, we respond accordingly in social interaction. Our views influence how we interact with others and how we cognitively appraise that interaction. This means that women’s relatively frequent sexual submission and men’s relatively frequent sexual dominance could be understood as a result of perceived norm sending of partners through a social tuning process. These processes presumably work mostly in an automatic, subconscious way (rather than being an attentive and deliberate process). We know this because experimental studies have shown that the romantic and sexual context automatically activates gendered behavior relatively strongly (Marks, 2008; Hundhammer and Mussweiler, 2012; Fisher, 2013) and that sexual context cues can instantly activate a mental link with submission among women (Sanchez et al., 2006), as well as invoke aggressive behavioral tendencies in men (Mussweiler and Förster, 2000). In this way, it is possible that the SDS is ‘recycled in social interaction’, even if peoples’ individual attitudes do not (or no longer) necessarily reflect these attitudes. The value of these interpersonal processes when studying the SDS has also been noted in previous research (Marks, 2008). This could partly explain why sexual attitudes appear to have become increasingly egalitarian (Eaton and Rose, 2011; Emmerink et al., 2016a), whereas surveys of sexual behavior continue to demonstrate gender differences in terms of sexual initiative taking (Morokoff et al., 1997; Schalet, 2010; Vannier and O’Sullivan, 2011; De Graaf et al., 2012; Sanchez et al., 2012). Furthermore, in addition to gender and gendered partner characteristics, we believe that participant gender-typicality could influence sexual initiative taking. People are active agents with their own goals and belief system, also when it comes to gender and when gender-schemas become activated, so do gender self-schemas (Deaux and Major, 1987). Already in school-aged children we see that that being a gender-typical boy or girl is linked with gender-stereotypical thinking and behavior (Patterson, 2012). Although early behavior-based measures for gender-typicality have not always been predicative of gender-related behaviors (Deaux and Major, 1987), we use a newer non-behavior-based measure in this study. Therefore, because they presumably value gender-typicality most, we would expect especially strong social tuning among highly gender-typical respondents, compared to low gender-typical respondents.

Some earlier experiments point toward the validity of the social tuning hypothesis concerning gendered processes (Zanna and Pack, 1975; Sinclair et al., 2005). Zanna and Pack (1975) let female subjects describe themselves to a male partner who was either desirable or not and who’s stereotype of the ideal woman conformed very closely to the traditional female stereotype or its opposite. They found that when the partner was desirable, the subjects portrayed themselves as more or less conventional in terms of sex-role, depending upon whether the partner’s stereotypic view of women was traditional or not. Social tuning seemed to occur, but only when the partner was desirable. Sex differences thus seem to be sort of a self-fulfilling prophecy. Another series of experiments (Sinclair et al., 2005) firstly showed that when anticipating contact with a male believed to hold traditional stereotype-consistent views of women (though no actual contact was subsequently established), female participants with a high interest in making new social bonds rated themselves as more stereotypically feminine, whereas those less interested in bonding did not. When women with a high interest in making new social bonds thought that the male held counter-stereotypical views of women, on the other hand, they also rated themselves less stereotypically, whereas those less interested in bonding did not. In a second experiment, they showed, adding a behavioral measure (participants were told that they were about to have ‘a normal conversation’ with a male who was actually a confederate), that women also behaved in line with their (non-) stereotypical self-ratings (in line with the findings of the first experiment) (Sinclair et al., 2005).

Although these experiments showed evidence for gendered processes in social tuning, the sample sizes were fairly small, participants were females only, the experimental context was not overtly sexual, and participant gender-typicality (masculine and feminine characteristics) was only investigated as an outcome measure. These latter aspects, in particular, mean these studies are unable to answer questions regarding the SDS and its link with sexual initiative taking.

### The Present Study

We proposed an experimental vignette approach, which is particularly suitable for investigating a social tuning hypothesis as it allows for a controlled context, while enabling manipulation of a single situational cue, namely target partner-traditionality. An experimental approach is able to overcome some of the limitations imposed by previously widely used cross-sectional methods in SDS research (Crawford and Popp, 2003; Bordini and Sperb, 2013), by providing an insight into causality and curbing socially desirable answering tendencies. We used a young adult sample because these young people are still relatively new to romantic and sexual interactions, whereas their relationships, to a greater extent than in adolescence, are more likely to include sexual intercourse (Arnett, 2000). This last notion is of importance because we only wanted to include participants who were already sexually active.

In this study we used an experimental vignette approach to investigate whether young men and women would socially tune their expectations toward their own sexual initiative taking to target partner traditionality in line with the differential social norms proposed by the SDS. We hypothesized that:

(1) Women will indicate lower levels of sexual initiative taking and men higher levels of sexual initiative taking in response to a vignette presenting a gender-traditional dating partner, compared to men and women presented with an identical vignette without information concerning target traditionality.

Secondly, we hypothesized that especially strong social tuning would occur among highly gender-typical participants, compared to low gender-typical participants, i.e., that;

(2) Highly gender-typical women will indicate lower levels of sexual initiative taking and highly gender-typical men will indicate higher levels of sexual initiative taking in response to a vignette presenting a gender-traditional dating partner, compared to men and women low in gender-typicality. In response to a vignette without information concerning target traditionality, we expect the same pattern to be less pronounced.

## Materials and Methods

### Sample

This paper draws on data from the LISS (Longitudinal Internet Studies for the Social Sciences) panel administered by CentERdata [Tilburg University, The Netherlands]. The LISS panel is a representative sample of Dutch individuals who participate in monthly Internet surveys from the comfort of their own homes (in exchange for a small reward). The panel is based on a true probability sample of (approximately 5000) households drawn from the population register. Households without a computer and Internet access are provided these by LISS. A random selection of LISS panel members from those households was invited to participate in the study. The number of eligible candidates in each household varied, according to how many household members subscribe to the panel and whether they fit our age inclusion criterion. The specific sample included in this study thus consisted of a unique draw from the participants in the LISS panel. More information about the panel can be found on their website^1^. This study was granted ethical approval by the Ethics Committee of the Faculty of Social and Behavioral Sciences of Utrecht University [Reference: FETC15-003 (Vanwesenbeeck)]. Of the original sample of 414 young adults (aged 18–25), 29 did not sign the consent form and consequently did not take part in the study. A first filter question assessed sexual experience; ‘How many people have you (by approximation) had sex with in your lifetime?’. A definition of sex was given; By ‘sex’ we mean everything from feeling each other naked or caressing each other, to intercourse (penetration of the vagina or anus by the penis). Only participants who indicated that they had been sexually active at some point continued to the questionnaire. This led to the exclusion of another 76 participants. Although it would be interesting to study this topic among non-heterosexual participants in the future, in this study we chose to focus on heterosexual interactions, mainly because of the exploratory nature of the study as well as the strong tie of heteronormativity to heterosexuality. Consequently, people who indicated that they were mainly or exclusively attracted to their own sex, who indicated that they were attracted to both sexes equally and those who indicated that they were unsure of their sexual preference, were excluded from the analyses. This resulted in the exclusion of another 38 people. The final sample for analysis consisted of 271 heterosexual young adults (60.15% female) aged between 18 and 25 years (*M* = 21.19, *SD* = 2.79).

### Procedure

An experimental vignette approach was employed, using a mixed factorial design with three factors; participant gender (male vs. female), target gender-traditionality (no information vs. traditional) and participant gender-typicality (continuous variable consisting of separate scores on masculinity and femininity). Participants first read an introduction stating that the study would be about the thoughts and feelings surrounding sexuality of young people aged between 18 and 25 years. It also stated that they would be reading a fictional sexual scenario involving a new romantic partner and would be asked to indicate their behavioral expectations in that situation. After reading this introduction, they could indicate consent or no consent by ticking the appropriate box. They were also informed that even after ticking the ‘consent’ box, they could still stop participating at any time. Consenting individuals proceeded to a page instructing them to read very carefully a vignette describing a (relatively) new romantic partner and a sexual scenario. They were urged to project themselves into the situation described, disregarding any real-life romantic commitments they might have. Participants were randomly assigned to a vignette describing a gender-traditional opposite sex target, or an equivalent vignette without any information about gender-traditionality. At the end of the vignette, all participants read the same sexual scenario, in which they were asked to imagine themselves spending the night with the presented partner. The text for the partner vignettes and sexual scenario is presented in Supplementary Material (Appendix A) (translated for the purpose of international readership). Subsequently, participants filled in the main outcome measure assessing sexual initiative taking, followed by a manipulation check and questions about demographics. At the very end of the questionnaire, they filled in a measure for individual gender-typicality, after which they were debriefed by e-mail. The order of measures in the questionnaire is reflected in the description of the materials below.

### Materials

#### Target Gender-Traditionality Vignettes

The text for the partner vignettes (presented in Supplementary Material, Appendix A) was thoroughly piloted before using it in this study. The vignette described a relatively new romantic partner. Female participants were presented with a vignette describing a male partner and male participants were presented with a vignette describing a female partner. To strengthen affiliative motives (as well as to try to keep this constant), the partner was described as ‘attractive,’ as ‘someone of the same age,’ and as ‘someone who does not live far from you.’ The manipulation in the experimental condition consisted of a single sentence: ‘You have also noticed that his/her opinion on relationships between men and women is rather traditional.’ The traditional target vignette was identical to the no traditionality information vignette, except for the sentence on partner gender-traditionality. At the end of the vignette, all participants read the same sexual scenario, which communicated that there had been multiple dates already and that there was mutual consent for and interest in spending the night together.

#### Expectations Regarding Sexual Initiative Taking

Three items designed for this study assessed expectations regarding sexual initiative taking, in which participants were asked how likely they would be to ‘take the initiative to (French) kiss,’ ‘take the initiative to touch his/her body under his/her clothes, once you are already kissing’ and ‘take the initiative to go a step further yet (for example having sex).’ Responses ranged from ‘1 = (Highly) unlikely’ to ‘5 = Very likely.’ All items operated in the same direction, i.e., higher scores reflected more intent toward initiative taking. We obtained a Cronbach’s alpha of 0.85 for this measure. A mean score was calculated from the three items for use in the analyses.

#### Manipulation Check

Participants were asked to rate the partner in the scenario on how ‘traditionally masculine’ (for the vignette about a male only)/’traditionally feminine’ (for the vignette about a female only) they thought the person was on a scale from 1 = not at all to 6 = very much.

#### Attractiveness

Participants were also asked to rate the partner in the scenario on ‘attractiveness,’ because both theoretically and based on a previous study, this perception could influence their motivation to act in a certain way (Zanna and Pack, 1975). We assessed partner attractiveness by asking ‘How attractive do you think he/she is?’, on a scale ranging from 1 = not at all to 6 = very much.

#### Demographics

Participants indicated their age, gender, sexual orientation, religiousness and current relationship status.

#### Target Gender-Typicality

Gender-typicality was measured using the Gender Typicality Scale (GTS+), which assesses the degree of masculinity and femininity as separate subscales among both men and women (Altstötter-Gleich, 2004). The principle applied in construction of the GTS+ was derived from the Bem Gender Role Inventory (BSRI; Bem, 1974), which contains items that are equally socially desirable for both sexes but are seen as more typical for one of the two sexes. Masculinity and femininity are seen as distinct concepts on which a person can simultaneously score high or low (Bem, 1977). The masculinity subscale consisted of eight items (which were translated into Dutch for this study): decisive, assertive, confident, fearless, business-like, daunting, resolute, and willing to take risks. The femininity subscale also consisted of eight items (again translated into Dutch): warm-hearted, empathetic, romantic, sensitive, understanding, hearty, emotional, and sensual. Feminine and masculine items were presented in alternating order. Participants were asked to describe to what extent these characteristics accurately described them in daily life, answering on a scale from 1 = seldom to 4 = always. In this study, we obtained an alpha of 0.71 for the masculine characteristics and 0.78 for the feminine characteristics. A mean score was calculated from the respective eight characteristics for ‘masculinity’ and ‘femininity.’

### Statistical Analyses

All analyses were conducted in IBM SPSS Statistics 22. Preliminary analyses were performed to check whether the manipulation was successful and whether there were differences in perceived attractiveness and current relationship status between the vignettes. Subsequently, two hierarchical regression analyses were performed; one including masculinity, and one including femininity [because they are separate dimensions (Bem, 1977)], both with sexual initiative taking as the outcome variable. In all hierarchical regression analyses target gender-traditionality, participant gender, and participant gender-typicality (i.e., either masculinity or femininity) were added in the first step, two-way interactions between these variables in the second step and their three-way interaction in the third step.

## Results

### Preliminary Analyses

We conducted preliminary *t*-tests to examine whether the man and woman described in the experimental vignette were indeed assessed as more gender-traditional compared to the man and woman described in the control vignette. The manipulation of traditionality seemed to be successful, as both the male and female traditional partner were, respectively, rated as significantly more ‘traditionally masculine’ (*M* = 4.36, *SD* = 0.89) and ‘traditionally feminine’ (*M* = 4.49, *SD* = 0.85) compared to the control condition [Male *M* = 3.88, *SD* = 0.77, *F*(1,107) = 8.86, *p* < 0.01; Female *M* = 4.14, *SD* = 0.85, *F*(1,162) = 6.23, *p* < 0.05].

To control for possible effects of the manipulation on perceived partner attractiveness, we tested the effect of the two partner vignettes on perceived attractiveness. A preliminary analysis showed that women who were assigned to the traditional partner vignette (*M* = 4.64, *SD* = 0.76) perceived their partner as significantly less attractive compared to the women who were assigned the control vignette [*M* = 4.87, *SD* = 0.63, *F*(1,162) = *p* < 0.05]. No differences emerged in perceived attractiveness among men assigned to the different vignettes [Traditional Female *M* = 5.02, *SD* = 0.51; Control Female *M* = 4.95, *SD* = 0.47, *F*(1,108) = 0.57, *p* = 0.45]. Based on the significant differences in the female sample, perceived attractiveness was added as a control variable in all hierarchical regression analyses in the first step.

### Regression Analysis with Masculinity

**FIGURE 1:**
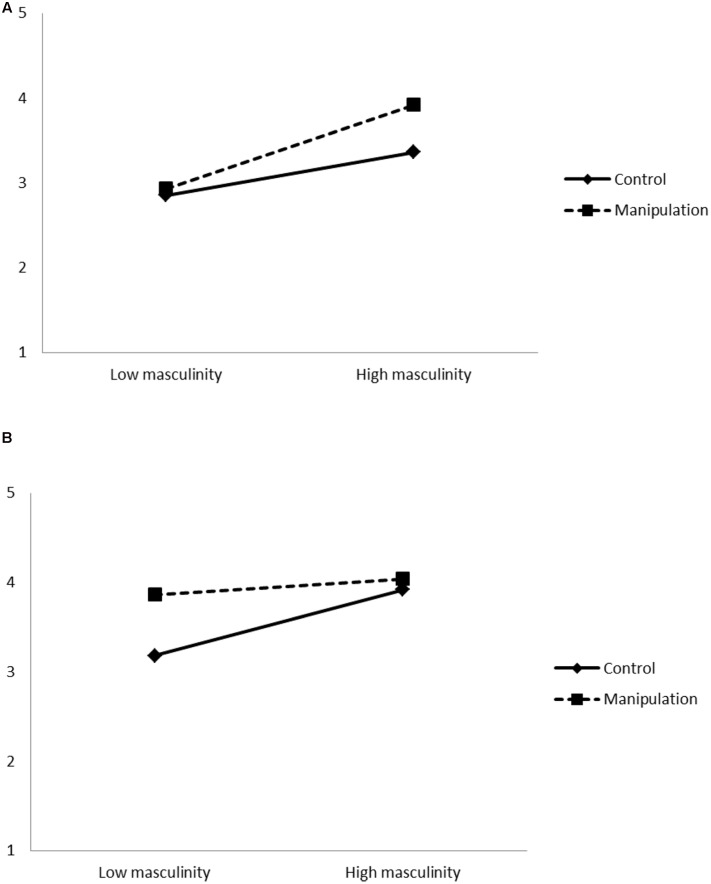
**(A)** Three-way interaction between participant gender, target gender-traditionality and participant gender-typicality (masculinity), on sexual initiative taking displayed separately for young women. **(B)** Three-way interaction between participant gender, target gender-traditionality and participant gender-typicality (masculinity), on sexual initiative taking displayed separately for young men.

A hierarchical regression analysis was conducted, with participant gender, condition and masculinity as the independent variables, perceived attractiveness as a control variable, and sexual initiative taking as the outcome (see **Table 1**). As preliminary analyses confirmed that there were no outliers and no assumptions were violated, we proceeded with entering variables in three steps. The overall model test was significant [*F*(8,259) = 8.92, *p* < 0.001, *R*^2^ = 0.22]. In the first step, gender, degree of masculinity and perceived attractiveness were significant predictors, but condition was not. Compared to men, women reported lower levels of sexual initiative taking. Higher scores on masculinity and higher perceived attractiveness were both related to higher scores on sexual initiative taking. In the second step, no significant effects emerged for the added two-way interactions. However, in the third step, the added three-way interaction between gender, condition and degree of masculinity emerged as significant. The three-way interaction is plotted separately for men and women in **Figures 1A,B**.

**Table 1:** Summary of hierarchical regression analysis for variables predicting sexual initiative taking, including masculinity (*N* = 271).

Variable	Step 1				Step 2				Step 3			
	*B*	*SE B*	β	*p*	*B*	*SE B*	β	*p*	*B*	*SE B*	β	*p*
Target gender-traditionality	0.165	0.104	0.088	0.116	-0.007	0.166	-0.004	0.967	0.056	0.166	0.030	0.737
Participant gender (Male = 1)	-0.425	0.108	-0.224	0.000	-0.583	0.158	-0.308	0.000	-0.579	0.157	-0.305	0.000
Participant gender-typicality (masculinity)	0.538	0.128	0.237	0.000	0.522	0.236	0.230	0.028	0.867	0.276	0.382	0.002
Perceived attractiveness	0.285	0.082	0.198	0.001	0.290	0.082	0.202	0.000	0.295	0.082	0.206	0.000

Target traditionality × participant gender					0.289	0.216	0.148	0.181	0.258	0.214	0.132	0.230
Target traditionality × participant masculinity					-0.012	0.258	-0.004	0.962	-0.734	0.400	-0.232	0.068
Participant gender × participant masculinity					0.049	0.262	0.016	0.852	-0.561	0.368	-0.187	0.128

Target traditionality × gender × masculinity									1.219	0.520	0.298	0.020

*R*^2^ change step 1	0.193^∗∗∗^											
*R*^2^ change step 2 two-way Interactions					0.006							
*R*^2^ change step 3 three-way Interactions									0.017^∗^			

*Participant gender-traditionality (masculinity) was entered as a mean centered variable. In *post hoc* analyses we additionally checked the effect of religiousness and relationship status. Religiousness was a significant predictor (less sexual initiative taking was present among religious, compared to non-religious individuals), but did not alter the other relationships found. Relationship status was not a significant predictor. ^∗^*p* < 0.05, ^∗∗∗^*p* < 0.001*.

Follow-up simple interaction effect analysis showed a significant two-way interaction among men [*F*(2,267) = 3.30, *p* < 0.05] (**Figure 1B**), but not among women [*F*(2,267) = 0.76, *p* = 0.468] (**Figure 1A**). Consecutive simple effect analysis showed that for men higher scores on masculinity were related to higher scores on sexual initiative taking only in the control condition.

### Regression Analyses with Femininity

A hierarchical regression analysis was conducted, with participant gender, condition and femininity as the independent variables, perceived attractiveness as a control variable, and sexual initiative taking as the outcome (see **Table 2**). As preliminary analyses confirmed that there were no outliers and no assumptions were violated, we proceeded with entering variables in three steps. The overall model test was significant [*F*(4,263) = 5.77, *p* < 0.001, *R*^2^ = 0.15]. In the first step, gender and perceived attractiveness were significant predictors, but degree of femininity and condition were not. Compared to men, women reported lower levels of sexual initiative taking. Higher scores on perceived attractiveness were related to higher scores on sexual initiative taking. In the second step, no significant effects emerged for the added two-way interactions. In the third step, the added three-way interaction between gender, condition and degree of femininity was also non-significant.

**Table 2:** Summary of hierarchical regression analysis for variables predicting sexual initiative taking, including femininity (*N* = 271).

Variable	Step 1				Step 2				Step 3			
	*B*	*SE B*	β	*p*	*B*	*SE B*	β	*p*	*B*	*SE B*	β	*p*
Target gender-traditionality	0.183	0.107	0.098	0.090	0.008	0.173	0.004	0.964	-0.017	0.177	-0.009	0.925
Participant gender (Male = 1)	-0.542	0.116	-0.286	0.000	-0.701	0.169	-0.370	0.000	-0.701	0.169	-0.370	0.000
Participant gender-typicality (femininity)	0.139	0.121	0.070	0.250	0.239	0.214	0.119	0.265	0.320	0.251	0.160	0.203
Perceived attractiveness	0.301	0.085	0.210	0.000	0.309	0.086	0.215	0.000	0.311	0.086	0.217	0.000

Target traditionality × participant gender					0.301	0.229	0.153	0.191	0.307	0.230	0.157	0.182
Target traditionality × participant femininity					-0.169	0.242	-0.059	0.484	-0.336	0.360	-0.117	0.352

Participant gender × participant femininity					-0.040	0.244	-0.014	0.870	-0.186	0.339	-0.067	0.583

Target traditionality × gender × femininity									0.304	0.487	0.076	0.533
*R*^2^ change step 1	0.144^∗∗∗^											
*R*^2^ change step 2 two-way interactions					0.006							
*R*^2^ change step 3 three-way interactions									0.001			

*Participant gender-traditionality (femininity) was entered as a mean centered variable. In *post hoc* analyses we additionally checked the effect of religiousness and relationship status. Religiousness was a significant predictor (less sexual initiative taking was present among religious, compared to non-religious individuals), but did not alter the other relationships found. Relationship status was not a significant predictor ^∗∗∗^*p* < 0.001*.

## Discussion

In this study we investigated whether young men and women would socially tune their sexual initiative taking to perceived partner gender-traditionality in line with the differential social norms proposed by the SDS. The hypothesized interaction between participant gender, target gender-traditionality and participant gender-typicality was found, but the direction of the effect did not match our expectations. Analyses showed that not the gender-typical, but the less gender- typical men were more likely to initiate sexual contact in the experimental, compared to the control condition; men low in masculine characteristics showed higher initiative taking in response to a gender-traditional female. This points to the conclusion that, at least among men, less gender-typical individuals employ more social tuning, whereas more gender-typical individuals seem to adhere to their gender-typical behavior regardless of their partner’s gender-traditionality. This finding warrants some discussion.

Contrary to our hypotheses, the results refuted the idea that men’s self-proclaimed masculinity would further spur sexual assertiveness when they were confronted with a gender-traditional female, as this only seemed to be the case among those non-typical for their gender (scoring low on masculine characteristics). Only less gender-typical men who scored low on masculinity showed higher sexual assertiveness in the form of increased sexual initiative taking in the experimental compared to the control condition. It could be that these young men who score low on masculinity either feel they have to compensate more compared with more masculine young men, or simply that they dare to be bolder when they expect to encounter a gender-traditional, i.e., relatively non-assertive woman. For them, encountering a gender-traditional female partner might present an opportunity for agency (hence the increase in the experimental condition) that might be lacking when they are confronted with a partner about whom they have no information on which to base their expectations.

Also contrary to our hypotheses was that no such effect was seen among women. Although here too it appeared that less gender-typical individuals (who rated themselves higher on masculine characteristics) tended to initiate sexual activity more when confronted with a seemingly gender-traditional target, this interaction did not reach significance. Therefore, based on this study, we cannot argue that social tuning occurs among women. Increased importance of individual agency and personal responsibility in contemporary Western society may also explain these results. It appears that the norms concerning agency are even more complicated today for women than they were in the past, as women are not stigmatized simply for being sexually active, but for being sexually active and sub-assertive, as well as for being sexually passive and sub-assertive (Bay-Cheng, 2015). This ambiguity may have led the results among women to be less pronounced compared to men, resulting in the non-significance of the interaction.

Taken together these findings refuted our expectation that stronger social tuning would be seen among highly gender-traditional respondents, at least among men, because particularly less gender-typical respondents (i.e., men low in masculinity) indicated higher sexual initiative taking in response to a gender-traditional target. This leads us to believe that, whereas highly gender-typical men may invariably respond in a gender-typical way, less gender-typical men tend to employ more social tuning and appear to be more fluid in their behavioral response; relatively less assertive men initiate sex more easily (and take their opportunities) notably with women expected to be relatively sub-assertive. Respondents relatively gender-typical themselves, i.e., highly masculine men, seemed to be less sensitive to partner characteristics and be rooted firmer in their own (gendered) behavior.

It should be noted, however, that the discussed results only concerned the masculinity factor of our gender-typicality measure and the analysis including the degree of femininity yielded null-results. Whereas masculinity and femininity are separate concepts that are in no way mutually exclusive (Bem, 1974, 1977), the null-results concerning femininity do warrant some discussion. We could speculate that this is because masculine characteristics are generally more valued in society than feminine characteristics and therefore have a larger effect in the analyses. However, further research would be needed to enable any definite conclusions to be drawn concerning the role played by feminine characteristics in sexual situations.

Lastly, although no separate hypothesis was formulated on this matter, we found that overall, women indicated lower levels of sexual initiative taking, compared to men, regardless of the vignette they were assigned to. As stated in the introduction, this is in line with earlier studies, which commonly find relatively frequent sexual initiative taking by men and relatively frequent sexual compliance by women (Morokoff et al., 1997; Vannier and O’Sullivan, 2011; De Graaf et al., 2012; Sanchez et al., 2012). Although these patterns could in part be the result of innate gender differences, gender differences are often found to be very small (Vanwesenbeeck, 2009). Some research even suggests that it is not as much biological sex, but gender orientation/gender identity that is responsible for the differences that are found (Vanwesenbeeck, 2009). It therefore seems more likely that gender differences in sexual initiative taking have been heavily influenced by decades of double standards in sexuality for men and women (Crawford and Popp, 2003; Sanchez et al., 2012; Bordini and Sperb, 2013). We believe that future studies could provide valuable information that could be used to narrow the gender gap in sexual initiative taking (and sexual assertiveness in general).

### Limitations and Implications

In this study we experimentally placed people in a (fictional) sexual scenario with a gender-traditional vs. a gender-neutral partner using a vignette. The experimental method is still relatively uncommon in this field, but has several advantages over other methods (e.g., cross-sectional studies), in that it provides an insight into causality and reduces the risk of socially desirable responses. When choosing an experimental method, there is always a trade-off between ensuring that a manipulation in a vignette is not too overt, while still being strong enough to have an effect. Particularly regarding the null-findings for femininity, it may very well be that the degree of femininity does have effects in sexual situations, but that the employed vignette and outcome measure were substantively less related to this concept than with the degree of masculinity. As expectations of masculinity include agency, whereas expectations of femininity generally have more to do with communion, results might have shown effects of femininity instead of masculinity, if we had chosen to investigate for example sexual communication (feeling comfortable to communicate sexual wishes and limits) (Santos-Iglesias et al., 2014). Future studies could further the knowledge regarding this interesting notion.

Secondly, in this study, we chose to investigate sexual initiative taking as ‘the ability to initiate sexual contact in the sexual situation,’ the situation being a given because of the sexual scenario that was offered. In doing so, we were unable to account for selection effects on gendered characteristics; participants were not given a choice as to whether they would ‘meet’ the traditional or the neutral partner. In real life, these selection effects might imply that individuals with differing views on gender roles do not seek each other out at all. Although we did control for perceived attractiveness in the analyses, we cannot rule out the possibility that individuals with highly discrepant views might form a somewhat unlikely pairing and that, in that sense, our experiment was less able to adequately reflect real-life processes. However, we do not believe that this compromised the external validity of the study. The scenario is still one that could easily occur in real life. It is possible to imagine meeting a highly gender-traditional partner, even if it is not a person you would necessarily seek out yourself.

In a similar vein, it could be the case that results were additionally influenced by perceptions of individual attractiveness that the respondents have of themselves. Although we did not measure this in the current study, it would be good to control for this variable in future work.

What adds to this is that when we first meet someone, gender norms are presumably communicated mostly through non-verbal and non-overt verbal cues, i.e., we read ‘between the lines’ to ascertain (amongst other things) how gender-traditional someone might be. Most of the times an individual will simply not have the kind of straightforward information that was presented in the vignette. An interesting avenue for future studies might therefore be to investigate the ways in which we communicate gender norms and explore how subsequent selection of partners takes place and how this affects social tuning processes. This implies that future studies could benefit from broadening the conceptualization of sexual (or romantic) initiative taking. Shedding light on social initiative taking and making advances in the romantic context (e.g., looks and smiles), preceding sexual contact and sexual initiative taking, could further our knowledge on how social tuning works. We believe that experimental work could be part of this exploration, but also note that field studies in which couples are asked to reflect on their (sexual) relationship, might be useful.

Thirdly, we do not know for sure what people were thinking about when they read ‘You have also noticed that his/her opinion on relationships between men and women is rather traditional.’ It could be argued that participants understood this as ‘no sex before marriage’ instead of interpreting it as the role you play in sex, disregarding such things as religious beliefs and other contextual factors. However, because participants were instructed to disregard any real life commitments and because the sexual scenario emphasized mutual desire and consent, i.e., willingness to engage in sexual contact, we believe to have surpassed interference by such beliefs. Moreover, we additionally checked *post hoc* whether religiousness and current relationship status were significant predictors of sexual initiative taking. While this was indeed the case for religiousness only (being religious predicted lower sexual initiative taking), controlling for this variable did not change the other relationships in the model. Future experimental studies could aim to shed more light on this to make the understanding of the concept more explicit.

Fourthly, this study being a first experimental investigation of the social tuning process in sexuality, we only investigated a heterosexual sample. Of course, heteronormative social concepts also affect non-heterosexual relationships (Szymanski and Henrichs-Beck, 2014). Future studies may want to look at processes involving social tuning in sexuality for sexual minority groups as well. This might even yield information that is helpful in understanding how heteronormativity works in both heterosexual and non-heterosexual populations.

Fifthly, we were unable to account for the possible influence of evolutionary and genetic differences within or between individual respondents. It is, however, possible that physical gender differences in for example hormones (e.g., initiative taking as a product of ovulation) and genetic make-up exert some influence on our results. Many studies show, however, that there are more similarities than differences between men and women in these aspects and that the differences are often small (Hyde, 2005; Buss and Schmitt, 2011).

Lastly, we wish to note the cultural embeddedness of these results. A different cultural setting may yield completely different results. Therefore, it would be interesting to examine a variety of cultural settings in the future, notably in one with a less liberal cultural climate concerning sexuality.

## Conclusion

Our findings appear to support the understanding of sexual behavior as a ‘now you see it, now you don’t’ (Deaux and Major, 1987) phenomenon, as the situational display of sexual assertiveness in the form of initiative taking seems to be dependent on a multitude of gendered characteristics. Although the SDS seems to tie in to this complex interplay of gendered characteristics, more research is needed to be able to draw more definite conclusions. In that sense, the findings of this study could serve as a starting point for the further development of experimental investigations regarding the gendered nature of sexual initiative taking, as well as sexual assertiveness in general. Moreover, it could serve as a starting point for the investigation of similar questions in different (e.g., non-heterosexual) samples. The study justifies further exploration of sexual behavior using experimental methods and it seems that vignettes can play a significant role in this process.

The results of this study provided partial evidence to support the social tuning hypothesis in relation to sexual initiative taking, but also indicated that these processes are more intricate than expected. Our analyses suggest that sexual initiative taking is probably as dependent on subjects’ gendered characteristics, notably the degree of masculinity, as on subjects’ gender and the partner’s gender-traditionality. Contrary to our expectations and only among men, did less gender-typical individuals employ more social tuning, whereas more gender-typical men seemed to remain (sub-)assertive regarding initiative taking regardless of the partner they encounter. We conclude that at least among men, it appears that those less gender-typical seem to be more flexible in attuning to partner characteristics. It is possible that this fluidity extends to other (situational) factors, which is a notion that deserves more attention in future research. In practice, our findings imply that both gender-typical and less gender-typical young people could benefit from sexual education including sexual assertiveness as a topic, as well as from interventions aimed at increasing sexual assertiveness.

## Ethics Statement

This study received ethical approval from the Ethics Committee of the Faculty of Social and Behavioural Sciences of Utrecht University [Reference: FETC15-003 (Vanwesenbeeck)]. Research involving human participants. All procedures performed were in accordance with the ethical standards of the institutional and/or national research committee and with the Ethical Principles of Psychologists and Code of Conduct (APA, 2010). Informed consent and debriefing: Informed consent was obtained from all individual participants included in the study. Participants were aware of their right to cease participation at any time. They were ensured that they would remain anonymous and that their answers would be handled confidentially. Although no deception was part of the experimental manipulation, participants were debriefed through e-mail directly following their participation explaining the design and purpose of the study. Included in the debriefing was contact information of the researchers in case participants had any additional questions. Phone numbers for independent Dutch (sexuality) helplines were also provided, in case participants felt the need to talk about their sexual experiences or other sexual issues.

## Author Contributions

PE, RV, TTB, and IV contributed in study design and the development of the materials for the study. PE conducted the research, collected and analyzed the data and was responsible for the main write-up of the manuscript. RV, TTB, and IV contributed as co-authors in the editing and re-writing of the manuscript.

## Conflict of Interest Statement

All authors currently work at Utrecht University. The fourth author IV also works at Rutgers, a non-profit expert center for sexual and reproductive health and rights.

## Footnotes

1 http://www.lissdata.nl/lissdata/
