# Supplementary material for: The Impact of Personal Gender-Typicality and Partner Gender-Traditionality on Taking Sexual Initiative: Investigating a Social Tuning Hypothesis
Source: Front Psychol. 2017 Feb 1;8:107. doi: 10.3389/fpsyg.2017.00107 (PMC5285358; doi:10.3389/fpsyg.2017.00107)
Supplement: Supplementary file 1 [file Data_Sheet_1.docx]

**Appendix A**

**Partner Vignette Text**

Some weeks ago a friend of yours set you up on a date with a very nice young woman/young man*. She/He is the same age as you are and lives not far from you. Your friend told you: ‘I’m positive you will like her/him. You would be a great match’.

Since then, you have been on several dates with her/him; you ‘click’ together amazingly well, and on top of that she/he is incredibly attractive! She/He clearly shows that she/he really likes you, too.

You have had some very enjoyable dates with her/him when you have had great fun. You have got to know her/him as a person who is actively involved in social media and who follows the news. You have also noticed that her/his views on relationships between men and women are fairly traditional.

Imagine that you have been out with her/him on a date again. You have had a great evening. There is sexual tension in the air and you both feel like spending the night together.

*Female participants were presented with a scenario about a young man, and male participants were presented with a scenario about a young woman. In the control condition, the underlined sentence was not presented. The vignette has been translated for the purpose of international readership. The original Dutch wording can be obtained from the authors on request.
